# Effect of Supplementation of Quercetagetin on the Antioxidant Function, Liver Mitochondrial Function and Gut Microbiota of Broilers at High Stocking Density

**DOI:** 10.3390/ani15030398

**Published:** 2025-01-31

**Authors:** Shuo Yang, Zixuan Su, Min Huo, Cuihong Zhong, Fangfang Wang, Yongying Zhang, Yaqi Song, Yuxiang Shi

**Affiliations:** College of Life Science and Food Engineering, Hebei University of Engineering, Handan 056038, China; yangshuo7336@163.com (S.Y.); szx0501@163.com (Z.S.); huomin5224@163.com (M.H.); zhongcuihong20070@163.com (C.Z.); wf28888@126.com (F.W.); zhangyongying1972@163.com (Y.Z.); songyaqi14@163.com (Y.S.)

**Keywords:** quercetagetin, broiler chicken, antioxidant capacity, mitochondrial function, gut microbiota

## Abstract

This study investigates the effects of quercetagetin (QG) supplementation on the antioxidant capacity, liver mitochondrial function, and cecal microbiota of broilers raised under high-density conditions. A 2 × 2 factorial design with 144 one-day-old WOD168 broilers, which were allocated to two stocking densities (LD: 4 birds per cage, equivalent to 11.1 birds per square meter; HD: eight birds per cage, equivalent to 22.2 birds per square meter) and two levels of dietary supplementation of QG (0 and 20 mg/kg). At the conclusion of day 21, broilers of similar body weights were randomly allocated into four groups (22 to 42 d): control (CON), QG treatment (QG), high stocking density (HSD), and high stocking density with QG supplementation (H_QG). The results demonstrated that HD groups significantly reduced broiler growth performance, including body weight (BW), average daily gain (ADG), and average daily feed intake (ADFI) (*p* < 0.05). Additionally, HD groups increased serum stress hormone levels (CORT and ACTH), pro-inflammatory cytokines (IL-1β and IL-6) (*p* < 0.05), while decreasing liver antioxidant enzyme activities (GSH-Px, T-SOD), serum CAT and T-SOD activities, and mitochondrial function (GSH, complex I-III, ATP contents) (*p* < 0.05). However, dietary supplementation with 20 mg/kg QG significantly alleviated the negative effects induced by HSD, restoring growth performance, stress hormone levels, immune parameters, and liver antioxidant and mitochondrial function. Moreover, QG supplementation markedly improved cecal microbiota composition, enhancing gut health. Correlation analysis revealed a strong association between microbial composition and overall broiler health, indicating that gut microbiota plays a critical role in mediating these beneficial effects. In conclusion, QG exhibits protective effects against oxidative stress, mitochondrial dysfunction, and gut microbiota imbalance induced by high-density rearing, suggesting its potential as a functional feed additive to improve broiler health under intensive farming conditions.

## Simple Summary

This study highlights the potential of quercetagetin (QG) as a functional feed additive to mitigate the adverse effects of high stocking density on broiler health. QG supplementation improved growth performance, reduced stress hormone levels, and enhanced liver antioxidant capacity and mitochondrial function. Additionally, QG positively influenced the cecal microbiota, promoting gut health. These findings suggest that QG could be an effective strategy to improve nutrient utilization, stress resilience, and overall health in broilers under intensive farming conditions, offering a cost-effective approach to enhance production efficiency and welfare in poultry systems.

## 1. Introduction

Intensive feeding practices are increasingly common in the broiler industry. While high stocking density (HSD) improves yield per unit area, it also leads to oxidative stress and immune suppression, which can impair broiler health and performance [1]. HSD triggers the overproduction of reactive oxygen species (ROS) and disrupts the antioxidant defense system, leading to oxidative damage in key organs such as the liver [2] and intestines [3,4]. The liver, as the primary metabolic organ, is particularly vulnerable to oxidative damage under HSD conditions, which negatively affects energy metabolism and nutrient processing [5]. Additionally, oxidative stress alters gut microbiota composition, further compromising intestinal health and reducing growth performance [6]. Therefore, addressing oxidative stress is essential for improving both the health and productivity of broilers.

Mitochondria are essential for cellular processes such as adenosine triphosphate (ATP) synthesis, metabolic regulation, calcium homeostasis, and redox balance [7,8,9,10]. Proper mitochondrial function is crucial for cellular homeostasis, and its dysfunction, often due to excessive ROS, can cause structural damage, metabolic impairment, and altered cell death regulation, especially under oxidative stress conditions such as HSD [11,12]. To counteract this, mitochondria rely on intrinsic antioxidant defense mechanisms, including the thioredoxin 2 (Trx2) and glutathione (GSH) systems, to neutralize ROS and preserve function [13,14]. Mitochondrial biogenesis-the synthesis of new mitochondria and enhancement of existing networks to meet energy demands is regulated by key factors such as peroxisome proliferator-activated receptor gamma coactivator 1-alpha (PGC-1α), nuclear respiratory factors 1 and 2(NRF1, NRF2), and mitochondrial transcription factor A (TFAM). These factors promote mitochondrial function by driving mtDNA replication and mitochondrial protein synthesis [15]. In response to oxidative stress, cells activate mitochondrial biogenesis to repair damaged mitochondria and restore function. PGC-1α plays a pivotal role in this adaptive process by upregulating antioxidant enzymes, such as superoxide dismutase (SOD) and glutathione peroxidase (GPx), thereby strengthening cellular defenses against oxidative damage [16]. This intricate interplay between mitochondrial biogenesis, mitochondrial function, and oxidative stress serves to maintain mitochondrial integrity and mitigate oxidative damage, particularly in broilers.

Natural plant extracts have gained attraction for their potent antioxidant, anti-inflammatory, and immune-modulating properties. Among these, quercetagetin (QG), a flavonoid compound found in various plants, exhibits strong antioxidant and immune-modulating effects. Studies have indicated that QG can effectively scavenge excessive ROS, mitigate cellular oxidative damage, promote growth and enhance immune function, thereby improving the stress resistance and production performance of broilers [17]. However, research on its effects on liver mitochondrial function and gut microbiota in HSD conditions remains limited. Therefore, this study aims to assess the effects of dietary QG on growth performance, liver mitochondrial function, and gut microbiota in broilers under HSD conditions, with a focus on its potential to alleviate oxidative stress.

## 2. Materials and Methods

### 2.1. Experimental Animals and Design

The experiment utilized a 2 × 2 factorial design with 144 one-day-old WOD168 broilers (purchased from Hebei Yuko Poultry Industry Co., Ltd., Handan, China), which were allocated to two stocking densities (LD: 4 birds per cage, equivalent to 11.1 birds per square meter; HD: eight birds per cage, equivalent to 22.2 birds per square meter) and two levels of dietary supplementation of QG (0 and 20 mg/kg). The broilers were housed in a three-tier cage system, with each cage measuring 60 cm × 60 cm, resulting in a cage area of 0.36 m^2^ per cage. The birds were reared uniformly from days 1 to 21 within these cages. At the conclusion of day 21, broilers of similar body weights were randomly allocated to one of four treatment groups, with six replicates per group. These groups were as follows: The control group (CON), maintained at a stocking density of four birds per cage (equivalent to 11.1 birds per square meter), and fed a basic diet; the QG group, maintained at a stocking density of four birds per cage (equivalent to 11.1 birds per square meter), with the basic diet supplemented with 20 mg/kg of QG (Chenguang Biotech Group Co., Ltd., Handan, China, purity > 85%); the high-density group (HSD), maintained at a stocking density of eight birds per cage (equivalent to 22.2 birds per square meter), and also fed the basic diet; and the high-density QG group (H_QG), maintained at a stocking density of eight birds per cage (equivalent to 22.2 birds per square meter), with the basic diet supplemented with 20 mg/kg of QG. Based on our previous research findings, the inclusion level of QG in the diet was established [18]. The experimental duration was from day 22 to day 42. The diets, based on corn and soybean meal, were formulated according to the Feeding Standard for Chickens in China (NY/T 33-2004), ensuring that the nutritional needs of the broilers were adequately met (Chinese Ministry of Agriculture) [19]. The composition and primary nutritional components of the diets are detailed in Table 1.

### 2.2. Rearing Management

The broilers were housed in a chicken house that had been thoroughly cleaned and disinfected, ensuring adequate ventilation to maintain a purified environment. Throughout the experimental period, standard management practices were adhered to, providing the broilers with ad libitum access to feed and water. The immunization schedule followed established guidelines. The chicken house temperature was maintained at 34 °C for the initial three days, followed by a gradual reduction of 2–3 °C per week from day four onwards, stabilizing at 22 °C until the end of the experiment. An incremental lighting system, as described by Oliveira et al. [20], was implemented. This system provided 23 h of light and 1 h of darkness during the first three days (days 1–3), followed by 10 h of light per day from days 4 to 21, and then returned to 23 h of light per day from days 22 to 42. The color of light was white and its intensity was 4 W/m^2^. The relative humidity within the chicken house was consistently maintained at levels between 50% and 70%.

### 2.3. Growth Performance Measurement

Body weight (BW) and feed intake were recorded weekly for each replicate group of broiler chicks on days 22 and 42 of the experiment. Using these data, the average daily feed intake (ADFI), average daily gain (ADG), and feed-to-gain ratio (F/G) were calculated for the period between days 22 and 42.

### 2.4. Sample Collection

On day 42, a single broiler from each replicate group, with a BW approximating the group average, was selected for sample collection. Blood was collected via the wing vein and then centrifuged at 3000× *g* for 15 min to isolate the serum. The resulting supernatant was collected and stored at −20 °C for subsequent measurements of serum immune and antioxidant parameters. Following blood collection, the experimental birds were euthanized, and samples of the liver and cecum were obtained. The left lobe of the liver was placed in sterile EP tubes for the determination of immune and antioxidant parameters. Meanwhile, the right lobe and cecal contents were collected in sterile EP tubes, rapidly frozen in liquid nitrogen, and stored at −80 °C for future RNA extraction and analysis of the gut microbiota.

### 2.5. Serum Hormone Measurement

Quantitative measurements of serum cortisol (CORT), adrenocorticotropic hormone (ACTH), insulin-like growth factor I (IGF-I), and growth hormone (GH) were performed using enzyme-linked immunosorbent assay (ELISA) kits, following the manufacturer’s instructions meticulously (Jiancheng Bioengineering Institute, Nanjing, China).

### 2.6. Measurement of Immune and Antioxidant Parameters in Serum and Liver

The activities of glutathione peroxidase (GSH-Px), catalase (CAT), and total superoxide dismutase (T-SOD), as well as the malondialdehyde (MDA) content, were assessed in serum and liver samples. Additionally, the concentrations of interleukin-1 beta (IL-1β), IL-6, immunoglobulin A (IgA), IgG, and IgM in serum and liver tissues were measured. All assays were conducted using kits provided by Nanjing Jiancheng Bioengineering Institute, with procedures strictly following the manufacturer’s instructions.

### 2.7. Preparation of Liver Mitochondria and Measurement of Antioxidant Parameters

The preparation of mitochondria from broiler liver tissues was performed according to a modified protocol previously described by Michael et al. [21]. Briefly, the liver samples were collected and rinsed with cold normal saline (0.9%) to remove residual blood and debris, after which they were minced into small pieces. The minced liver tissues were then homogenized in an ice-cold isolation buffer composed of 10 mM Tris-HCl, 250 mM sucrose, and 1 mM EDTA, with the pH adjusted to 7.4 using Tris. The homogenate was centrifuged at 800× *g* for 5 min at 4 °C to remove cellular debris and nuclei. The supernatant was collected and subjected to a second centrifugation at 12,000× *g* for 15 min at 4 °C to pellet the mitochondria. The resulting mitochondrial pellet was washed twice by resuspension in a fresh isolation buffer and centrifuged again under the same conditions. Finally, the purified mitochondria were resuspended in the isolation buffer and stored at −80 °C for further analysis. The contents of GSH and the activity of manganese superoxide dismutase (MnSOD), as well as the levels of MDA, were measured using kits from Nanjing Jiancheng Bioengineering Institute.

### 2.8. Measurement of Liver Mitochondrial Respiratory Chain Complexes and ATP Contents

The activities of mitochondrial respiratory chain complexes I-IV and ATP contents in mitochondria were assessed using commercial kits from SinoBestBio (Beijing, China), following the manufacturer’s guidelines.

### 2.9. Total RNA Isolation and Gene Expression Analysis

Total RNA was extracted from liver tissue using Trizol reagent (Carlsbad, CA, USA), and the purity and concentration of RNA were assessed using a Nano-Drop 2000 spectrophotometer (Thermo Fisher Scientific, Waltham, MA, USA). An OD260 /OD280 ratio between 1.8 and 2.0 indicated good RNA quality suitable for subsequent experiments. cDNA synthesis was performed using the PrimeScript™ RT Reagent Kit with gDNA Eraser (Takara Bio Inc., Dalian, China). Real-time quantitative PCR was conducted using the SYBR^®^ Premix ExTaq™ II kit (Tli RNaseH Plus, Dalian, China)) to assess the relative expression of genes. All reagents were obtained from Takara Bio Inc. (Dalian, China). Quantitative Real-Time PCR was utilized to quantify the copy number of mtDNA as previously described. Total DNA was extracted from liver samples using a genomic DNA extraction kit (TakaRa Biotechnology Co., Dalian, China). Equal aliquots of DNA were amplified and quantified through real-time PCR analysis. The mtDNA displacement loop (D-loop) and β-Actin served as markers for mtDNA and nuclear DNA, respectively, and the 2^-ΔΔCt^ method was employed for calculations. Detailed primer information for liver-related genes is provided in Table 2, with all primers synthesized by Sangon Biotech (Shanghai, China) Co., Ltd.

### 2.10. Analysis of Cecal Microbiota

Cecal contents from the right side were collected into 1.5 mL cryotubes, immediately flash-frozen in liquid nitrogen, and stored at −80 °C until further analysis. Microbiota profiling was conducted using high-throughput sequencing of the 16S rRNA gene.

### 2.11. Statistical Analysis

The data were analyzed using a two-factor analysis of variance (ANOVA) with SPSS 22.0 software to assess the main effects and interaction effects of QG and HD, based on a 2 × 2 experimental design. Statistical significance was defined as *p* < 0.05, highly significant differences were indicated by *p* < 0.01, and *p* > 0.05 was considered as no significant difference.

## 3. Results

### 3.1. Growth Performance

The effects of QG on the growth performance of broilers under high-density rearing conditions are presented in Table 3. On day 42, broilers in the HD groups showed a significant reduction in BW, ADG, and ADFI compared to the LD groups (*p* < 0.05). Additionally, a significant interaction between dietary QG and HD was observed for ADG (*p* < 0.05).

### 3.2. Serum Hormone Measurement

The effects of QG on stress hormone levels in broilers reared under high-density conditions are presented in Table 4. On day 42, serum concentrations of CORT and ACTH were significantly elevated in the HD groups compared to the LD groups (*p* < 0.05), while GH levels were significantly reduced (*p* < 0.05). Dietary supplementation with QG significantly reduced the serum levels of CORT and ACTH in the broilers compared to the basic diet without DQ (*p* < 0.05). Furthermore, a significant interaction between dietary QG and HD conditions was observed in serum ACTH levels (*p* < 0.05).

### 3.3. Serum and Liver Immunity Parameters

The effects of QG on serum cytokine and immunoglobulin levels in broilers reared under high-density conditions are summarized in Table 5. Compared to the LD groups, the HD groups exhibited significantly increased serum levels of IL-1β and IgG (*p* < 0.01) and IgM levels (*p* < 0.05). In the liver, HD conditions significantly increased IL-6 levels (*p* < 0.01) and elevated both IL-1β and IgM levels (*p* < 0.05). Dietary supplementation with QG significantly reduced liver IL-1β levels (*p* < 0.05). Additionally, a significant interaction between dietary QG and HD conditions was observed in serum IgG levels (*p* < 0.05), as well as in liver IL-1β, IL-6, and IgM levels (*p* < 0.01).

### 3.4. Serum and Liver Antioxidant Parameters

The effects of QG on oxidative status indicators in broilers reared under high-density rearing conditions are presented in Table 6. Compared to the LD groups, the HD groups exhibited significantly reduced serum CAT and T-SOD activities (*p* < 0.05), along with decreased liver GSH-Px and T-SOD activities (*p* < 0.05). In contrast, MDA content was significantly increased in the HD groups (*p* < 0.05). Dietary supplementation with QG significantly increased serum T-SOD activity (*p* < 0.05), as well as liver GSH-Px and T-SOD activities (*p* < 0.05), while markedly reducing MDA content (*p* < 0.05) compared to the basic diet. Additionally, significant interactions between dietary QG and HD conditions were observed in liver GSH-Px and T-SOD activities (*p* < 0.01), as well as in MDA content (*p* < 0.05).

### 3.5. Antioxidant Parameters of Liver Mitochondria

The effects of QG on the antioxidant parameters of liver mitochondria in broilers under high-density rearing conditions are presented in Table 7. Compared to the LD groups, the HD groups exhibited a significant decrease in GSH content in the liver mitochondria (*p* < 0.05) and a marked increase in MDA content (*p* < 0.01). Dietary supplementation with QG significantly increased MnSOD activity (*p* < 0.01) and significantly reduced MDA content (*p* < 0.05) compared to the basic diet. Furthermore, a significant interaction between dietary QG and HD conditions was observed in liver mitochondrial GSH and MDA contents (*p* < 0.05).

### 3.6. Mitochondrial Respiratory Chain Complex Activity

The effects of QG on the activities of mitochondrial respiratory chain complexes in the livers of broilers reared under high-density rearing conditions are presented in Table 8. Compared to the LD groups, the HD groups exhibited a significant reduction in the activity of complex I and complex III in the liver mitochondria (*p* < 0.01) and a notable decrease in complex II activity (*p* < 0.05). Dietary supplementation with QG significantly increased the activity of complex I and complex III (*p* < 0.05) compared to the basic diet. Furthermore, an interaction between dietary QG and HD was observed in the activity of complexes I and III in the liver mitochondria (*p* < 0.05).

### 3.7. Mitochondrial mtDNA and ATP Content in the Liver

**Figure 1:**
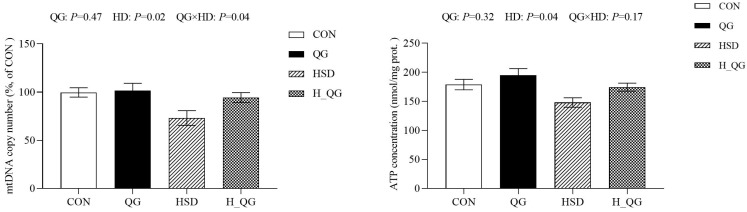
Effects of quercetagetin (QG) on mitochondrial mtDNA and ATP content in the liver of broilers under high-density rearing conditions. Note: LD, four birds per cage, equivalent to 11.1 birds per square meter; HD: eight birds per cage, equivalent to 22.2 birds per square meter. CON, with a stocking density of four birds per cage, fed a basic diet; QG, with a stocking density of four birds per cage, supplemented with 20 mg/kg of QG in the basic diet; HSD, with a stocking density of eight birds per cage, fed the basic diet; H_QG, with a stocking density of eight birds per cage, supplemented with 20 mg/kg of QG in the basic diet. Values represent the means ± SE and *n* = 6.

The effects of QG on mitochondrial *mtDNA* and ATP content in the liver of broilers reared under high-density rearing conditions are shown in Figure 1. Compared to the LD groups, the HD groups exhibited a significant reduction in both the *mtDNA* copy number and ATP content in the liver (*p* < 0.05). Furthermore, an interaction between dietary QG and HD conditions was observed in the liver *mtDNA* copy number (*p* < 0.05).

### 3.8. Expression of Genes Related to Mitochondrial Biogenesis

**Figure 2:**
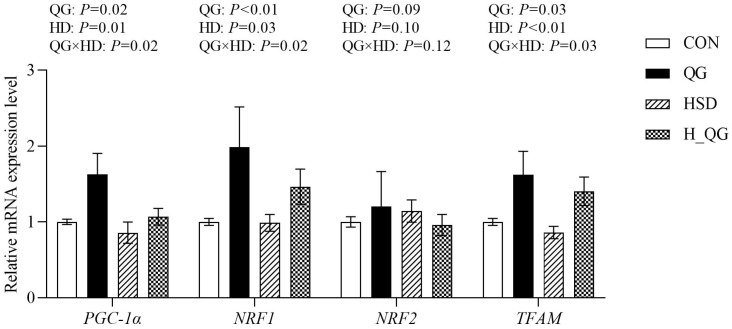
The effect of quercetagetin (QG) on mRNA expression of mitochondrial biogenesis-related genes in the liver of broilers under high stocking density. Note: LD, four birds per cage, equivalent to 11.1 birds per square meter; HD: eight birds per cage, equivalent to 22.2 birds per square meter. *PGC-1α* peroxisome proliferator-activated receptor γ coactivator, *NRF1* Nuclear respiratory factor 1, *NRF2* Nuclear respiratory factor 2, *TFAM* Mitochondrial transcription factor A. CON, with a stocking density of four birds per cage, fed a basic diet; QG, with a stocking density of four birds per cage, supplemented with 20 mg/kg of QG in the basic diet; HSD, with a stocking density of eight birds per cage, fed the basic diet; H_QG, with a stocking density of eight birds per cage, supplemented with 20 mg/kg of QG in the basic diet. Values represent the means ± SE and *n* = 6.

The effects of QG on the mRNA expression of genes related to mitochondrial biogenesis in the liver of broilers under high-density rearing conditions are illustrated in Figure 2. Compared to the LD groups, HD groups exhibited a significant reduction in the liver mitochondrial mRNA expression levels of *PGC-1α* and *NRF1* (*p* < 0.05), as well as a marked decrease in *TFAM* expression (*p* < 0.01). Dietary supplementation with QG significantly increased the mRNA expression levels of *PGC-1α* and *TFAM* (*p* < 0.05) and notably elevated *NRF1* expression (*p* < 0.01). Furthermore, an interaction between dietary QG and HD was observed in the liver mitochondrial mRNA expression levels of *PGC-1α*, *NRF1*, and *TFAM* (*p* < 0.05).

### 3.9. Expression of Antioxidant-Related Genes in Liver Mitochondria

**Figure 3:**
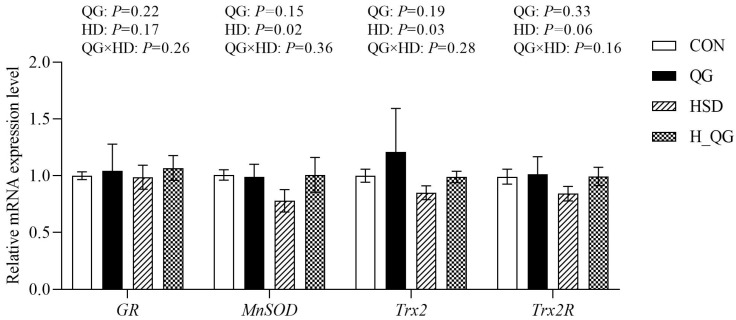
The effect of quercetagetin (QG) on mRNA expression of antioxidant function-related genes in the liver mitochondria of broilers under high stocking density. Note: LD, four birds per cage, equivalent to 11.1 birds per square meter; HD: eight birds per cage, equivalent to 22.2 birds per square meter. *GR* Glutathione reductase, *MnSOD* Manganese superoxide dismutase, *Trx2* Thioredoxin 2, *Trx2R* Thioredoxin 2 reductase. CON, with a stocking density of four birds per cage, fed a basic diet; QG, with a stocking density of 4 birds per cage, supplemented with 20 mg/kg of QG in the basic diet; HSD, with a stocking density of 8 birds per cage, fed the basic diet; H_QG, with a stocking density of 8 birds per cage, supplemented with 20 mg/kg of QG in the basic diet. Values represent the means ± SE and *n* = 6.

The effects of QG on the mRNA expression of mitochondrial antioxidant-related genes in the liver of broilers under high-density rearing conditions are shown in Figure 3. Compared to the LD groups, the HD groups exhibited a significant reduction in the mRNA expression levels of liver mitochondrial *MnSOD* and *Trx2* (*p* < 0.05).

### 3.10. Cecal Microbial Community Diversity

**Figure 4:**
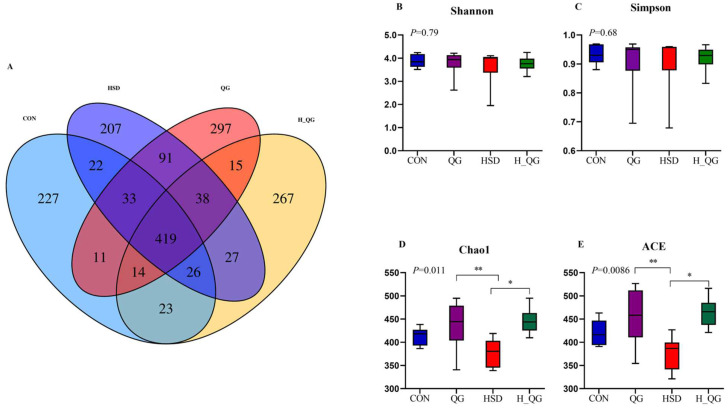
Effects of quercetagetin (QG) on OTU clustering analysis and α-diversity of cecal microbiota in broilers under high stocking density Note: (**A**), Venn plot of OUT distribution of cecal microbiota; (**B**), Shannon index; (**C**), Simpson index; (**D**), Chao1 index; (**E**), ACE index. * Indicated significant difference (*p* < 0.05); ** Indicated significant difference (*p* < 0.01).

The analysis of cecal microbial diversity in broilers under high-density rearing conditions supplemented with QG is shown in Figure 4. Cluster analysis of effective sequences from each group, conducted at a 97% similarity threshold, yielded a total of 1717 OTUs among the four groups, as shown in Figure 4A. The CON group exhibited 227 OTUs, accounting for 13.22% of the total OTUs; the HSD group had 207 OTUs, representing 12.06% of the total; the QG group contained 297 OTUs, comprising 17.30% of the total; and the H_QG group had 267 OTUs, making up 15.55% of the total. As presented in Figure 4D,E, the addition of QG to the diet significantly increased the Chao1 index and ACE index under high-density rearing conditions (*p* < 0.05). In contrast, the HSD group significantly decreased both the Chao1 index and ACE index compared to the CON group (*p* < 0.05).

**Figure 5:**
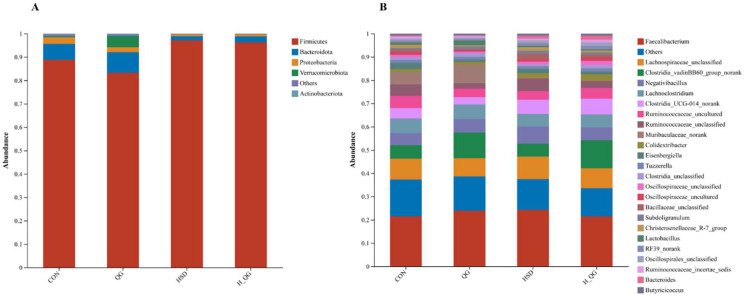
Effects of quercetagetin (QG) on the microbial composition of the cecum of broilers under high stocking density. Note: (**A**). The relative abundance of microbial community at the phylum level; (**B**). The relative abundance of microbial community at the genus level.

The results of 16S rRNA sequencing revealed that at the phylum level (Figure 5A and Table 9), the top four most abundant taxa across the groups were Firmicutes, Bacteroidota, Proteobacteria, and Verrucomicrobiota, with Firmicutes and Bacteroidota being the dominant phyla, collectively accounting for over 90%. Compared to the LD groups, the HD groups showed a significant increase in the abundance of Firmicutes (*p* < 0.05) and a significant decreas in the abundance of Bacteroidota (*p* < 0.05). At the genus level (Figure 5B), HD groups exhibited a significant increase in the abundance of *Clostridia_UCG-014_norank* and *Ruminococcaceae_unclassified* (*p* < 0.05). Additionally, dietary supplementation with QG significantly increased the abundance of *Clostridia_vadinBB60_group_norank* (*p* < 0.05).

**Figure 6:**
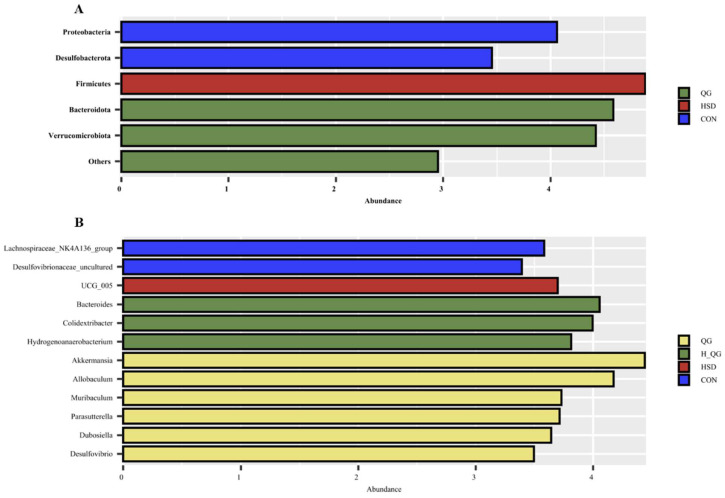
Effects of quercetagetin (QG) on LEfSe analysis of cecal microbiota in broilers under high stocking density. Note: (**A**). Phylum-level Linear Discriminant Analysis (LDA) with a score threshold of >3; (**B**). Genus-level Linear Discriminant Analysis (LDA) with a score threshold of >3.

Linear discriminant analysis (LDA = 3) effect size (LEfSe) algorithm was employed to analyze the taxonomic abundance of the microbial community. At the phylum level (Figure 6A), the abundance of Proteobacteria and Desulfobacterota increased in the CON group. In the HSD group, Firmicutes showed a significant increase, while Bacteroidota and Verrucomicrobiota were significantly enriched in the QG group. At the genus level (Figure 6B), the CON group exhibited significant enrichment of *Lachnospiraceae_NK4A136_group* and *Desulfovibrionaceae_uncultured*. In the HSD group, the abundance of UCG_005 significantly increased. The H_QG group displayed significant increases in the abundance of *Bacteroides*, *Colidextribacter*, and *Hydrogenoanaerobacterium*. Conversely, in the QG group, the abundances of *Akkermansia*, *Allobaculum*, *Muribaculum*, *Parasutterella*, *Dubosiella*, and *Desulfovibrio* significantly elevated.

### 3.11. Correlation Analysis of Growth Performance, Immune and Oxidative Function Indicators with Cecal Microbiota

**Figure 7:**
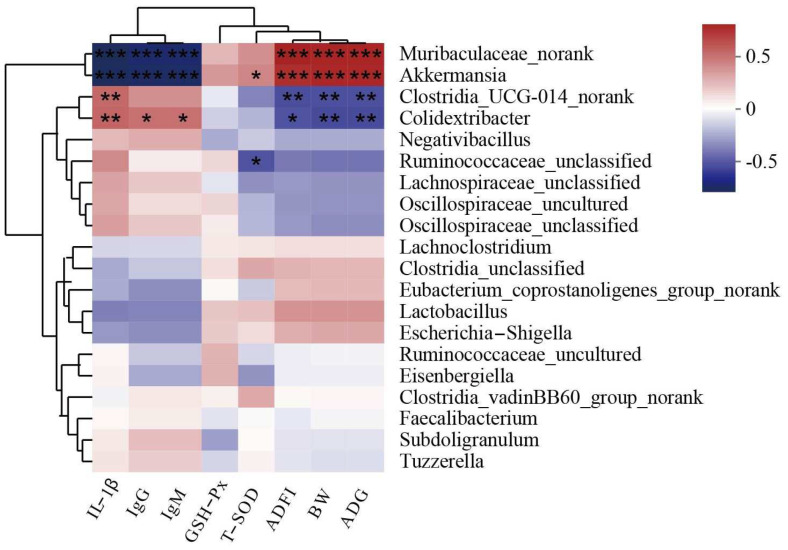
Correlation analysis between cecal microbiota and growth performance, serum immunity, and antioxidant capacity in broilers (Pearson correlation analysis)**.** Note: * Indicates significant difference (*p* < 0.05); **, *** Indicates extremely significant difference (*p* < 0.01).

Figure 7 illustrates the correlation analysis between the proportions of intestinal microbiota at the genus level and growth performance, serum immunity, and antioxidant function among the four groups. The results indicate a significant negative correlation between IL-1β, IgG, and IgM with *Muribaculaceae_norank* and *Akkermansia*. Specifically, IL-1β showed a significant positive correlation with *Clostridia_UCG-014_norank* and *Colidextribacter*, while IgG and IgM were significantly positively correlated with *Colidextribacter*. Additionally, ADFI, BW, and ADG exhibited significant positive correlations with *Muribaculaceae_norank* and *Akkermansia*, and significant negative correlations with *Clostridia_UCG-014_norank* and *Colidextribacter*. Furthermore, T-SOD was significantly positively correlated with *Akkermansia*, whereas it showed a significant negative correlation with uncultured *Ruminococcaceae*.

## 4. Discussion

In the poultry industry, the growth performance of broilers is intimately tied to economic benefits, with key performance indicators including ADG, ADFI, and F/G. Research has shown that HSD adversely affects broiler growth performance, not only impeding growth but also potentially elevating morbidity and mortality rates [22,23]. In HSD farming environments, restricted space and excessive population density can elicit oxidative stress responses in broilers, adversely impacting their health status and production performance. Specifically, HSD conditions promote the accumulation of ROS in the body, thereby disrupting the balance of the antioxidant defense system. This disruption, in turn, adversely affects growth and metabolic processes. As a result, broilers raised under HSD conditions typically exhibit increased F/G, along with suppressed ADG and reduced BW. These findings agree with previously published studies, which also highlight the detrimental effects of overcrowding on broiler growth performance [24,25,26,27]. These conditions contribute to oxidative stress, which adversely affects the birds’ physiological state by disrupting nutrient absorption and utilization, ultimately impairing growth performance. To address the negative impacts of high-density rearing, researchers have proposed various management strategies, including genetic improvements, dietary adjustments, and environmental controls [28]. However, these measures often require significant costs and technical support, making the search for low-cost and effective solutions particularly important. In this context, QG, a natural antioxidant, has garnered widespread attention for its potential in alleviating oxidative stress and improving broiler growth performance [29]. In this study, QG did not significantly enhance BW, ADG, or ADFI of broilers. However, a significant interaction between dietary QG and HD rearing was observed on broiler ADG, suggesting that QG may mitigate the negative effects of oxidative stress under crowded conditions. HSD can elevate oxidative stress, impairing growth, but QG’s antioxidant properties may help alleviate these effects by reducing cellular damage. Future studies should explore the underlying mechanisms of QG’s action, including its impact on stress biomarkers, immune responses, and mitochondrial function, to optimize its use in poultry production.

HSD not only affects the growth performance of broilers but also impairs the endocrine system, disrupting the circulating levels of hormones such as corticosterone [24]. Serum CORT and ACTH are common indicators reflecting the physiological stress state in livestock and poultry. Studies have demonstrated that under stress conditions, cytokines such as IL-1β can penetrate the central nervous system, triggering the activation of the hypothalamic-pituitary-adrenal (HPA) axis. This activation results in a rapid elevation of ACTH and glucocorticoid (GC) levels in the bloodstream [30]. The generated ACTH further stimulates the adrenal cortex to synthesize and release steroids, promoting the conversion of cholesterol to CORT, thereby increasing the concentration of CORT in serum. Elevated serum CORT levels trigger an increase in the catabolism of nutrients to meet the demands of normal physiological functions, resulting in altered nutrient allocation. Research indicates that HSD significantly increases CORT levels in broiler blood while decreasing feed intake, which subsequently affects daily weight gain, leading to oxidative stress in broilers [31]. The results of this experiment demonstrate that CORT and ACTH levels in the serum of broilers in the HD groups were significantly higher than those in the LD groups, indicating that HSD exacerbates oxidative stress responses in poultry, consequently slowing the growth and development of broilers. Additionally, the reduction in serum growth hormone (GH) and insulin-like growth factor I (IGF-I) levels is another important marker of stress response. GH stimulates the growth and differentiation of chondrocytes in poultry, while IGF-I, as a direct regulator of animal growth, stimulates the proliferation and differentiation of various cells, promotes protein synthesis, and contributes to the production of connective tissues and bone marrow; both hormones play crucial roles in the growth and development of poultry as well as feed conversion [32]. This study found that HD groups significantly reduced serum GH concentrations in broilers, suggesting that HSD may inhibit the growth performance of broilers by inducing stress responses. Notably, nutritional interventions can mitigate the negative effects of oxidative stress, including the abnormal elevation of serum corticosterone levels or the reduction of GH concentrations. This study demonstrated that QG supplementation significantly reduced the HSD-induced increase in ACTH, likely due to its antioxidant properties. QG scavenges ROS, which are known to activate the HPA axis and trigger excessive ACTH and cortisol production. Through the activation of the Nrf2 pathway, QG enhances the expression of antioxidant enzymes like SOD, CAT, and GPx, which help regulate ACTH secretion [33]. Additionally, QG hepatoprotective effects preserve liver function and reduce inflammation, supporting metabolic health and stress resilience, ultimately improving growth performance and feed efficiency in broilers [34].

The production of free radicals in animals is a continuous process, with the liver playing a crucial role as a primary site for the body’s antioxidant defenses [35]. The liver is one of the vital organs responsible not only for metabolic functions but also for detoxification, glycogen storage, and protein synthesis. Selvam et al. [36] previously reported that overcrowding leads to oxidative stress in broilers, as evidenced by decreased levels of GSH and increased levels of MDA in liver tissue. Similarly, research by Li et al. [23] demonstrated that on days 35 and 42, excessive crowding reduced serum activities of SOD and GSH-Px while elevating MDA levels in broilers. Our study found that HSD significantly decreased the activity of antioxidant enzymes (such as CAT and T-SOD) in both serum and liver while increasing the liver oxidative damage marker MDA content. These changes suggest that HSD exacerbates oxidative stress, adversely affecting the health of broilers. However, the dietary supplementation of QG under HSD significantly increased serum T-SOD activity and markedly reduced MDA levels in the liver. We hypothesize that QG effectively enhances the antioxidant capacity of broilers. This finding not only indicates the activation of the antioxidant enzyme system by QG but also supports its potential to improving the growth performance of broilers.

Previous research has indicated that rearing too many poultry in confined spaces can lead to physiological stress and impaired immune function [37]. Immunoglobulins are essential components of humoral immunity, and their levels reflect the immune function and physiological state of livestock. Low levels of immunoglobulins indicate reduced immunity, while high levels suggest a possible inflammatory state. Prior studies have shown that HSD can induce humoral immune responses in broilers, excessively activating the immune system and leading to significantly elevated serum immunoglobulin levels [38]. Our experimental results demonstrate that HD significantly elevated serum IgG and IgM levels in broilers and markedly increased liver IgM levels. This increase likely reflects an immune stress response triggered by HSD, stimulating plasma cells to secrete large amounts of immunoglobulins. However, under HSD conditions, the addition of QG significantly reduced serum IgG levels and decreased liver IgM levels, indicating a regulatory effect of QG on immune function in broilers under stress. Raising density can adversely affect the environment within the poultry house; HSD can deteriorate air quality, temperature, and humidity. Limited space for movement, restricted access to feed, and increased likelihood of stress due to hunger, injury, or fright all contribute to decreased immunity in animals. Das et al. [39] found that compared to normal rearing density (12 birds/m^2^), broilers in high-density rearing (20 birds/m^2^) had significantly elevated serum levels of IL-1β and IL-6. Our study demonstrates that HSD significantly elevated serum IL-1β and liver IL-6 levels in broilers, with a concurrent marked increase in liver IL-1β levels. This may reflect that in actual production settings, high-density rearing leads to overcrowding, potentially inducing localized specific immune responses that impair immune function in broilers. However, dietary supplementation with QG under HD conditions can significantly mitigate the abnormal increases in IL-1β and IL-6 levels. These results further indicate the potential role of QG in alleviating stress in broilers.

Mitochondria are essential for cellular energy production through oxidative phosphorylation, supporting key biological processes. In addition to their primary role in energy metabolism, increasing evidence suggests that mitochondria are involved in diverse physiological functions, such as programmed cell death, immune responses, autophagy, redox signaling, calcium homeostasis, and stem cell reprogramming [9,40]. The tricarboxylic acid (TCA) cycle generates NADH and FADH2, which fuel oxidative phosphorylation by serving as substrates for complexes I and II of the electron transport chain. Our study found that, compared to the LD groups, the activity of complexes I and III in the liver tissues of broilers in the HD groups was reduced, suggesting that complexes I and III are involved in the increased superoxide leakage during oxidative stress. However, dietary supplementation with QG significantly increased the activity of complexes I and III. These findings indicate that oxidative stress may stimulate ROS production by inhibiting the activity of complex I, while QG can significantly alleviate the reduction in complex I activity. Mitochondrial oxidative stress in muscle has been associated with inefficiency and poor feed utilization in broilers [41]. Our results suggest that mitochondrial oxidative damage caused by oxidative stress is at least partially responsible for the decline in growth performance in broilers. Research has shown that the activities of NADH dehydrogenase and cytochrome c oxidase decrease, with the latter’s decline linked to a shift in the redox balance of the electron transport chain toward excessive reduction, potentially leading to electron leakage and subsequent overproduction of ROS [42]. The decrease in electron transport chain activity signifies a decline in electrochemical potential or an increase in ROS levels. Moreover, we observed an increase in oxidative damage indicators in liver mitochondria, such as elevated MDA levels, reduced GSH content, and decreased ATP content, further supporting the impact of high-density rearing on mitochondrial function. According to Hirata et al. [43] research findings, external stimuli can inhibit the replication of mitochondrial DNA, reduce mitochondrial biogenesis, and ultimately alter mitochondrial quantities. Our findings revealed that the mitochondrial DNA replication was affected in the HD groups, where the *mtDNA* copy number can reflect the quantity of mitochondria. High-density rearing significantly disrupted the morphological structure of liver mitochondria in broilers and induced mitochondrial dysfunction. Notably, the addition of QG significantly alleviated these adverse effects. QG can regulate oxidative stress, improve mitochondrial biofunction, promote ATP synthesis, and enhance antioxidant capacity, thereby mitigating the damage to hepatic mitochondria caused by high-density rearing. This finding provides crucial evidence for further research into the potential applications of QG in improving the growth performance and health status of broilers.

Oxidative stress significantly impacts mitochondrial function in broilers, with PGC-1α serving as a critical transcription factor for mitochondrial biogenesis [44]. Under stress conditions, the expression of PGC-1α is downregulated, leading to impaired mitochondrial function. PGC-1α regulates mitochondrial biogenesis through the PGC-1α/NRF-1/TFAM signaling pathway. Specifically, NRF1, a key downstream effector of PGC-1α, controls the expression of genes essential for mitochondrial proliferation and function. Additionally, TFAM is crucial for the transcription and replication of mitochondrial DNA. A reduction in PGC-1α expression disrupts the activity of both NRF1 and TFAM, further exacerbating mitochondrial dysfunction and compromising cellular energy metabolism, particularly under oxidative stress conditions [11]. Our study results indicated that the expression of *PGC-1α*, *NRF1*, and *TFAM* genes in the liver mitochondria of broilers in the HD groups was significantly lower compared to the LD groups, leading to mitochondrial dysfunction. The primary cause of this dysfunction may be the decreased expression of PGC-1α, which results in reduced GSH levels, impaired antioxidant enzyme activity, and disruption of the cellular redox balance, ultimately causing mitochondrial dysfunction in the cells. Additionally, MnSOD, Trx2, and Trx2R in mitochondria play important roles in protecting cells against oxidative stress. MnSOD is the primary antioxidant enzyme within mitochondria, effectively scavenging superoxide anions, while Trx2 and its reductase Trx2R participate in cellular antioxidant defenses by regulating the activity of thioredoxins. The mitochondrial dysfunction caused by high-density rearing may impact the expression of MnSOD and Trx2 through the downregulation of PGC-1α, consequently reducing broilers’ resistance to oxidative stress [45]. Our results showed that the expression levels of MnSOD and Trx2 genes in the HD groups were significantly lower than those in the LD groups, further demonstrating the inhibitory effects of high-density rearing on mitochondrial antioxidant capacity. Notably, the supplementation of QG exhibited positive effects in alleviating the damage to mitochondrial function caused by high-density rearing. Our research indicates that QG can upregulate *PGC-1α* expression, promote mitochondrial biogenesis, and thus enhance cellular energy metabolism and antioxidant capacity. Furthermore, QG may improve the expression levels of *MnSOD* and *Trx2*, enhancing the resistance of broilers to oxidative stress. These findings suggest that QG, as a potential nutritional intervention strategy, can effectively mitigate the negative effects of high-density rearing on mitochondrial function in broilers, thereby promoting their health and growth.

The gut harbors a vast and complex microbial community that interacts closely with the host, influencing physiological health through involvement in metabolic, nutritional, and developmental processes [46]. The cecum serves as a site for food fermentation and further digestion; its closed end allows for prolonged retention of digesta, resulting in particularly rich and dense microbial communities [47]. Alpha diversity, as indicated by ACE, Chao1, Shannon, and Simpson indices, is an important measure of microbial diversity. This study found that in a high-density farming environment, the addition of QG to the diet significantly increased the Chao1 and ACE indices while having no significant effect on the Shannon and Simpson indices. This result suggests that the addition of QG may enhance gut microbial diversity, thereby helping to maintain ecological balance within the microbiota. Previous studies have indicated that the cecal microbiota is primarily composed of Firmicutes, Bacteroidetes, Actinobacteria, and Proteobacteria, which play crucial roles in maintaining intestinal microbial balance and promoting host health [48]. However, in modern intensive farming practices, the high-density rearing environment has become an unavoidable factor. Recent studies have shown that intensive farming methods, especially in high-density rearing systems, can increase the levels of airborne dust and bacteria, which pose significant risks to poultry health, particularly by negatively affecting their respiratory and digestive systems [49]. These conditions have been associated with increased incidences of respiratory infections and disruptions to the digestive system, leading to reduced growth performance and overall health [50,51]. Our study identified Firmicutes as the predominant bacterial phylum in the cecum of broilers, with a significant increase in its abundance in the HD groups compared to the LD groups. In contrast, the abundance of Bacteroidetes was significantly reduced under high-density rearing conditions. This finding indicates that the high-density rearing environment may promote the proliferation of Firmicutes, whereas the abundance of Bacteroidetes may be suppressed. As an important component of the cecal microbiota, the increase in Firmicutes abundance may reflect an adaptive response of the host to the stress of high-density rearing conditions. Firmicutes primarily participate in the fermentation of carbohydrates, producing beneficial metabolic by-products such as short-chain fatty acids (SCFAs). These by-products are crucial for maintaining gut barrier function, promoting nutrient absorption, and regulating immune responses. Thus, the increase in Firmicutes abundance in the HSD group may help broilers survive and adapt to adverse rearing environments. However, the reduction in Bacteroidetes abundance in the HSD group may indicate a decrease in gut microbial diversity, thereby increasing the risk of disease in poultry. Research has shown that Bacteroidetes have good fiber-degrading capabilities; when their abundance is high, the presence of fiber-degrading bacteria also increases [52]. Bacteroidetes play an important role in maintaining gut microbial community balance, improving host health, enhancing butyrate metabolism, strengthening gut barrier function, and maintaining homeostasis within the intestinal environment. At the genus level, the HD groups exhibited a significant increase in the abundance of *Clostridia*, further confirming the impact of high-density rearing on microbial populations. Our study observed that dietary supplementation with QG significantly increased the abundance of *Clostridia_vadinBB60_group_norank*. While previous research has not directly investigated *Clostridia_UCG-014_norank*, our findings suggest that this group may play a beneficial role in the intestinal microbiota of broilers. The increased abundance of *Clostridia_UCG-014_norank* could potentially contribute to the maintenance of intestinal microbiota balance. However, these conclusions are speculative and warrant further investigation to validate their role and function within the gut microbiome. Moreover, our study revealed a significant increase in the abundance of *Clostridia_UCG-014_norank* in the HD groups. This suggests that *Clostridia* may have diverse implications for intestinal health. On the one hand, *Clostridia* species are known to produce beneficial metabolites, including vitamins and SCFAs, which contribute to intestinal health and homeostasis. On the other hand, certain *Clostridia* species may also produce potentially harmful factors, such as toxins, which could pose a risk to poultry health [53]. Although *Clostridia is* not the dominant genus in the gut microbiota, it warrants further investigation due to its potential dual role in intestinal health.

We further elucidated the complex relationship between gut microbiota at the genus level and various performance metrics in broilers, including growth performance, serum immunity, and antioxidant function, through correlation analysis. Notably, significant negative correlations were observed between pro-inflammatory markers—specifically IL-1β, IgG, and IgM and beneficial genera such as *Muribaculaceae_norank* and *Akkermansia*. This suggests that increased inflammatory markers may negatively impact the abundance of these beneficial microbes, potentially compromising gut health and reducing growth performance. Conversely, IL-1β exhibited a significant positive correlation with *Clostridia_UCG-014_norank* and *Colidextribacter*, indicating that higher levels of this inflammatory cytokine are associated with an increase in certain potentially pathogenic bacteria, which could contribute to inflammatory responses and hinder nutrient absorption, thereby negatively affecting growth metrics. Similarly, IgG and IgM showed significant positive correlations with *Colidextribacter*, suggesting a compensatory immune response to this genus, though its exact role in gut health requires further investigation. Shterzer et al. [54] reported a positive correlation between the presence of *Muribaculaceae_norank* and key growth performance indicators in broilers. Their study highlighted the importance of specific gut microbiota in optimizing growth metrics. Our study found that growth performance metrics, including ADFI, BW, and ADG, exhibited positive correlations with *Muribaculaceae_norank* and *Akkermansia*. These findings suggest that the abundance of these beneficial microbial genera may enhance nutrient absorption and overall growth performance in broilers. In contrast, significant negative correlations between these growth metrics and *Clostridia_UCG-014_norank* and *Colidextribacter* reinforce the detrimental effects of these genera on broiler growth. The analysis of antioxidant function revealed that T-SOD was positively correlated with *Akkermansia*, suggesting a protective role in maintaining redox balance and antioxidant capacity. However, the negative correlation between T-SOD and *Ruminococcaceae_uncultured* indicates potential oxidative stress from specific microbial communities, adversely affecting the antioxidant defenses in broilers. The addition of QG to the diet plays a crucial role in modulating these microbial interactions and improving broiler health. By increasing the abundance of beneficial bacteria such as *Lactobacillus* and *Alistipes*, known producers of SCFAs, QG may help mitigate the inflammatory responses indicated by IL-1β and enhance overall microbial balance. SCFAs are essential for maintaining gut integrity and improving nutrient absorption, thereby contributing to better growth performance. Our study demonstrates that QG effectively modulates the composition of the gut microbiota, enhances antioxidant defense mechanisms, and mitigates mitochondrial oxidative damage, thereby promoting growth performance in broilers. These findings provide further evidence of the synergistic effects of QG. In conclusion, QG shows promise as a dietary supplement that not only supports intestinal microbial health but also enhances antioxidant capacity, ultimately leading to improved production outcomes in poultry farming. The synergistic effects of QG in promoting beneficial microbial populations and enhancing antioxidant capacity suggest its potential as a dietary supplement to improve broiler health and production outcomes. Future studies should focus on elucidating the specific molecular mechanisms through which QG influences microbial communities, inflammatory pathways, and oxidative stress, providing deeper insights into its potential applications in poultry management strategies.

## 5. Conclusions

In summary, the dietary supplementation of 20 mg/kg QG significantly enhances the immune function of broilers, improves their antioxidant capacity and effectively alleviates mitochondrial dysfunction in the liver under high-density rearing conditions. Additionally, QG markedly improves the composition of cecal microbiota. The results indicate that the cecal microbial community is correlated, to some extent, with the growth performance, immune responses, and antioxidant status of broilers. These findings provide a scientific basis for the future development of QG-based functional feed additives.

## Figures and Tables

**Table 1:** Composition and nutrient levels of the basal diet (as-fed basis), %.

Items	1 to 21 Days of Age	22 to 42 Days of Age
Ingredients		
Corn	52.50	58.80
Soybean meal	40.00	33.80
Soybean oil	3.00	3.00
Dicalcium phosphate	1.90	1.80
Limestone	1.08	1.22
Salt	0.37	0.37
Lysine	0.05	0.03
Methionine	0.19	0.07
Premix ^(1)^	0.80	0.80
Choline chloride	0.11	0.11
Total	100.00	100.00
Nutrient levels ^(2)^		
Metabolic energy (MJ/kg)	12.42	12.62
Crude protein	21.77	19.65
Calcium	1.00	1.02
Available phosphorus	0.44	0.42
Lysine	1.34	1.15
Methionine	0.55	0.40
Cystine	0.40	0.36

Note: ^(1)^ Premix provided the following per kilogram of diet: vitamin A 9000 IU, vitamin D_3_ 3000 IU, vitamin E 26 mg, vitamin K_3_ 1.20 mg, vitamin B_1_ 3.00 mg, vitamin B_2_ 8.00 mg, vitamin B_6_ 4.40 mg, vitamin B_12_ 0.012 mg, nicotinic acid 45 mg, folic acid 0.75 mg, biotin 0.20 mg, calcium pantothenate 15 mg, Fe 100 mg, Cu 10 mg, Zn 108 mg, Mn 120 mg, I 1.5 mg, Se 0.35 mg. ^(2)^ Crude protein was measured value, while others were all calculated values.

**Table 2:** Primer sequences and parameters.

Genes	Gene Bank No.	Primer Sequences, 5′-3′	Length (bp)
β-Actin	NM_205518	F-GCCAACAGAGAGAAGATGACAC	118 bp
		R-GTAACACCATCACCAGAGTCCA	
mtD-loop	XM_015291451.1	F-AGGACTACGGCTTGAAAAGC	198 bp
		R- CATCTTGGCATCTTCAGTGCC	
PGC-1α	AB170013.1	F-GACGTATCGCCTTCTTGCTC	157 bp
		R-CTCGATCGGGAATATGGAGA	
NRF1	NM_001030646.1	F-AAGAACACGGCGTGACTCAA	274 bp
		R-TCGCTTCCGTTTCTTACCCG	
NRF2	NM_001007858.1	F-GAGCCCATGGCCTTTCCTAT	212 bp
		R-CACAGAGGCCCTGACTCAAA	
TFAM	NM_204100.1	F-GTGAAAGCCTGGCGAAACTG	136 bp
		R- CACAGCTCAGGTTACACCGT	
GR	XM_040671422.1	F-TCCTGACTACGGCTTCGAGA	150 bp
		R-AACTTGCCGTAACCACGGAT	
MnSOD	NM_204211.2	F-GTTACAGCTCAGGTGTCGCT	115 bp
		R-CTCCTTTAGGCTCCCCTCCT	
Trx2	NM_001031410.1	F-AGTACGAGGTGTCAGCAGTG	141 bp
		R-CACACGTTGTGAGCAGGAAG	
Trx2R	NM_001122691.1	F-CCGGGTCCCTGACATCAAA	94 bp
		R-TAGCTTCGCTGGCATCAACA	

Note: F forward primer, R reverse primer, β-Actin beta-actin, PGC-1α peroxisome proliferator-activated receptor γ coactivator, NRF1 Nuclear respiratory factor 1, NRF2 Nuclear respiratory factor 2, TFAM Mitochondrial transcription factor A, GR Glutathione reductase, MnSOD Manganese superoxide dismutase, Trx2 Thioredoxin 2, Trx2R Thioredoxin 2 reductase.

**Table 3:** Effects of quercetagetin (QG) on growth performance of broilers under high-density rearing conditions.

Item	LD		HD		SEM	*p*-Value		
	CON	QG	HSD	H_QG		QG	HD	QG × HD
BW (kg)	1.58	1.63	1.50	1.55	0.02	0.36	0.02	0.88
ADG (g/d)	74.61	79.87	68.93	69.88	1.68	0.64	0.03	0.04
ADFI (g/d)	128.11	134.43	124.69	126.52	1.97	0.46	0.02	0.59
F/G	1.90	1.84	1.93	1.85	0.06	0.45	0.75	0.11

Note: BW body weight, ADG average daily gain, ADFI average daily feed intake, F/G feed-to-gain ratio. LD, four birds per cage, equivalent to 11.1 birds per square meter; HD: eight birds per cage, equivalent to 22.2 birds per square meter. CON, with a stocking density of four birds per cage, fed a basic diet; QG, with a stocking density of four birds per cage, supplemented with 20 mg/kg of QG in the basic diet; HSD, with a stocking density of eight birds per cage, fed the basic diet; H_QG, with a stocking density of eight birds per cage, supplemented with 20 mg/kg of QG in the basic diet. SEM = Standard Error of the Mean (*n* = 6), In peer data, no letters or the same letters indicate no significant difference (*p* > 0.05), and different lowercase letters indicate a significant difference (*p* < 0.05).

**Table 4:** Effects of quercetagetin (QG) on serum stress hormones in broilers under high-density rearing conditions.

Item	LD		HD		SEM	*p*-Value		
	CON	QG	HSD	H_QG		QG	HD	QG × HD
CORT (ng/L)	29.37	28.65	39.87	30.39	2.27	0.03	0.02	0.11
ACTH (pg/L)	20.77	17.30	31.25	19.11	2.21	0.04	0.03	0.03
IGF-I (ng/L)	40.31	36.40	38.70	37.15	2.52	0.33	0.89	0.67
GH (ng/L)	4.28	5.01	3.42	3.93	0.33	0.34	0.04	0.41

Note: CORT corticosterone, ACTH adrenocorticotropic hormone, IGF-I insulin-like growth factor I, GH growth hormone. LD, four birds per cage, equivalent to 11.1 birds per square meter; HD: eight birds per cage, equivalent to 22.2 birds per square meter. CON, with a stocking density of four birds per cage, fed a basic diet; QG, with a stocking density of four birds per cage, supplemented with 20 mg/kg of QG in the basic diet; HSD, with a stocking density of eight birds per cage, fed the basic diet; H_QG, with a stocking density of 8 birds per cage, supplemented with 20 mg/kg of QG in the basic diet. SEM = Standard Error of the Mean (*n* = 6). In peer data, no letters or the same letters indicate no significant difference (*p* > 0.05), different lowercase letters indicate a significant difference (*p* < 0.05).

**Table 5:** Effects of quercetagetin (QG) on cytokines and immunoglobulins in broilers under high-density rearing conditions.

Item	LD		HD		SEM	*p*-Value		
	CON	QG	HSD	H_QG		QG	HD	QG × HD
Serum								
IL-1β (pg/mL)	122.07	119.83	163.92	127.92	5.24	0.23	<0.01	0.30
IL-6 (pg/mL)	5.43	5.67	6.44	5.94	1.28	0.08	0.51	0.42
IgA (μg/mL)	57.53	58.21	63.38	56.97	4.43	0.32	1.54	0.23
IgG (μg/mL)	474.18	502.17	652.43	537.65	22.50	0.43	<0.01	0.04
IgM (μg/mL)	141.26	155.69	287.42	189.51	27.17	0.62	0.03	0.17
Liver								
IL-1β (pg/mg prot.)	15.62	14.71	32.95	13.45	3.23	0.04	0.03	<0.01
IL-6 (pg/mg prot.)	1.03	0.95	3.84	1.33	0.20	0.07	<0.01	<0.01
IgA (μg/mg prot.)	4.83	5.07	5.25	5.26	0.44	0.27	0.13	0.21
IgG (μg/mg prot.)	52.35	56.26	59.02	53.77	8.98	0.46	0.57	0.39
IgM (μg/mg prot.)	10.03	11.57	20.17	11.79	1.58	0.43	0.03	<0.01

Note: IL-1β interleukin-1 beta, IL-6 interleukin-6, IgA immunoglobulin A, IgG immunoglobulin G, IgM immunoglobulin M. LD, four birds per cage, equivalent to 11.1 birds per square meter; HD: eight birds per cage, equivalent to 22.2 birds per square meter. CON, with a stocking density of four birds per cage, fed a basic diet; QG, with a stocking density of four birds per cage, supplemented with 20 mg/kg of QG in the basic diet; HSD, with a stocking density of eight birds per cage, fed the basic diet; H_QG, with a stocking density of eight birds per cage, supplemented with 20 mg/kg of QG in the basic diet. SEM = Standard Error of the Mean (*n* = 6), In peer data, no letters or the same letters indicate no significant difference (*p* > 0.05), different lowercase letters indicate a significant difference (*p* < 0.05), and different uppercase letters indicate an extremely significant difference (*p* < 0.01).

**Table 6:** Effects of quercetagetin (QG) on oxidative function-related indicators in broilers under high-density rearing conditions.

Item	LD		HD		SEM	*p*-Value		
	CON	QG	HSD	H_QG		QG	HD	QG × HD
Serum								
GSH-Px (U/mL)	3957.4	3644.8	2981.8	3805.6	211.17	0.45	0.15	0.08
CAT (U/mL)	5.85	6.23	2.74	4.72	0.52	0.65	0.03	0.30
T-SOD (U/mL)	356.12	383.43	237.11	368.69	28.15	0.04	0.03	0.41
MDA (nmol/mL)	3.53	2.95	4.91	3.96	0.31	0.23	0.08	0.25
Liver								
GSH-Px (U/mg prot.)	88.73	141.67	58.49	73.75	9.15	0.02	0.03	<0.01
CAT (U/mg prot.)	9.17	8.75	8.64	7.83	0.55	0.38	0.42	0.13
T-SOD (U/mg prot.)	892.5	1422.9	593.5	764.7	107.4	0.04	0.02	<0.01
MDA (nmol/mg prot.)	1.78	2.37	6.11	1.85	0.82	0.04	0.02	0.02

Note: GSH-Px glutathione peroxidase, CAT catalase, T-SOD total superoxide dismutase, MDA malondialdehyde. LD, four birds per cage, equivalent to 11.1 birds per square meter; HD: eight birds per cage, equivalent to 22.2 birds per square meter. CON, with a stocking density of four birds per cage, fed a basic diet; QG, with a stocking density of four birds per cage, supplemented with 20 mg/kg of QG in the basic diet; HSD, with a stocking density of eight birds per cage, fed the basic diet; H_QG, with a stocking density of eight birds per cage, supplemented with 20 mg/kg of QG in the basic diet. SEM = Standard Error of the Mean (*n* = 6), In peer data, no letters or the same letters indicate no significant difference (*p* > 0.05), different lowercase letters indicate a significant difference (*p* < 0.05), and different uppercase letters indicate an extremely significant difference (*p* < 0.01).

**Table 7:** Effects of quercetagetin (QG) on antioxidant enzymes and metabolite levels in liver mitochondria of broilers under high-density rearing conditions.

Item	LD		HD		SEM	*p*-Value		
	CON	QG	HSD	H_QG		QG	HD	QG × HD
Liver mictochondria								
GSH (mg/g prot.)	13.57	15.62	8.47	7.69	0.67	0.42	0.02	0.04
MnSOD (U/mg prot.)	16.55	19.38	15.96	18.73	0.39	0.02	0.27	0.69
MDA (nmol/mg prot.)	2.46	1.98	7.35	4.22	0.19	0.04	<0.01	0.03

Note: GSH glutathione, MnSOD superoxide dismutase, MDA malondialdehyde. LD, 4 birds per cage, equivalent to 11.1 birds per square meter; HD: 8 birds per cage, equivalent to 22.2 birds per square meter. CON, with a stocking density of 4 birds per cage, fed a basic diet; QG, with a stocking density of 4 birds per cage, supplemented with 20 mg/kg of QG in the basic diet; HSD, with a stocking density of 8 birds per cage, fed the basic diet; H_QG, with a stocking density of 8 birds per cage, supplemented with 20 mg/kg of QG in the basic diet. SEM = Standard Error of the Mean (*n* = 6), In peer data, no letters or the same letters indicate no significant difference (*p* > 0.05), and different lowercase letters indicate a significant difference (*p* < 0.05).

**Table 8:** Effects of quercetagetin (QG) on the activity of respiratory chain complexes in liver mitochondria of broilers under high-density rearing conditions.

Item	LD		HD		SEM	*p*-Value		
	CON	QG	HSD	H_QG		QG	HD	QG × HD
Liver mictochondria								
Complex I (μmol NADH/min/mg prot.)	10.06	9.83	4.39	9.02	0.35	0.02	<0.01	0.03
Complex II (μmol NADH/min/mg prot.)	7.89	8.54	7.02	6.97	0.22	0.12	0.04	0.37
Complex III (μmol cytochrome c/min/mg prot.)	22.37	20.89	14.65	19.42	0.56	0.03	<0.01	0.01
Complex IV (μmol CoQH2/min/mg prot.)	47.33	45.09	38.76	42.15	1.45	0.16	0.09	0.16

Note: LD, four birds per cage, equivalent to 11.1 birds per square meter; HD: eight birds per cage, equivalent to 22.2 birds per square meter. CON, with a stocking density of four birds per cage, fed a basic diet; QG, with a stocking density of four birds per cage, supplemented with 20 mg/kg of QG in the basic diet; HSD, with a stocking density of eight birds per cage, fed the basic diet; H_QG, with a stocking density of eight birds per cage, supplemented with 20 mg/kg of QG in the basic diet. SEM = Standard Error of the Mean (*n* = 6), In peer data, no letters or the same letters indicate no significant difference (*p* > 0.05), and different lowercase letters indicate a significant difference (*p* < 0.05).

**Table 9:** The Effect of QG on the cecal microbial diversity of broilers under high stocking density.

Item	LD		HD		SEM	*p*-Value		
	CON	QG	HSD	H_QG		QG	HD	QG × HD
Phylum								
Firmicutes	0.89	0.83	0.97	0.96	0.15	0.07	0.03	0.28
Bacteroidota	0.07	0.08	0.02	0.03	0.06	0.42	0.02	0.12
Genus								
*Faecalibacterium*	0.19	0.21	0.22	0.20	0.11	0.79	0.94	0.65
*Lachnospiraceae_unclassified*	0.08	0.07	0.09	0.08	0.02	0.24	0.22	0.92
*Clostridia_vadinBB60_group_norank*	0.05	0.09	0.05	0.11	0.06	0.03	0.07	0.72
*Clostridia_UCG-014_norank*	0.04	0.03	0.06	0.06	0.03	0.77	0.02	0.35
*Ruminococcaceae_unclassified*	0.04	0.02	0.05	0.02	0.02	0.41	0.02	0.95

Note: LD, four birds per cage, equivalent to 11.1 birds per square meter; HD: eight birds per cage, equivalent to 22.2 birds per square meter. CON, with a stocking density of four birds per cage, fed a basic diet; QG, with a stocking density of four birds per cage, supplemented with 20 mg/kg of QG in the basic diet; HSD, with a stocking density of eight birds per cage, fed the basic diet; H_QG, with a stocking density of eight birds per cage, supplemented with 20 mg/kg of QG in the basic diet. SEM = Standard Error of the Mean (*n* = 6), In peer data, no letters or the same letters indicate no significant difference (*p* > 0.05), and different lowercase letters indicate a significant difference (*p* < 0.05).

## Data Availability

The data presented in this study are available upon request from the corresponding author.
